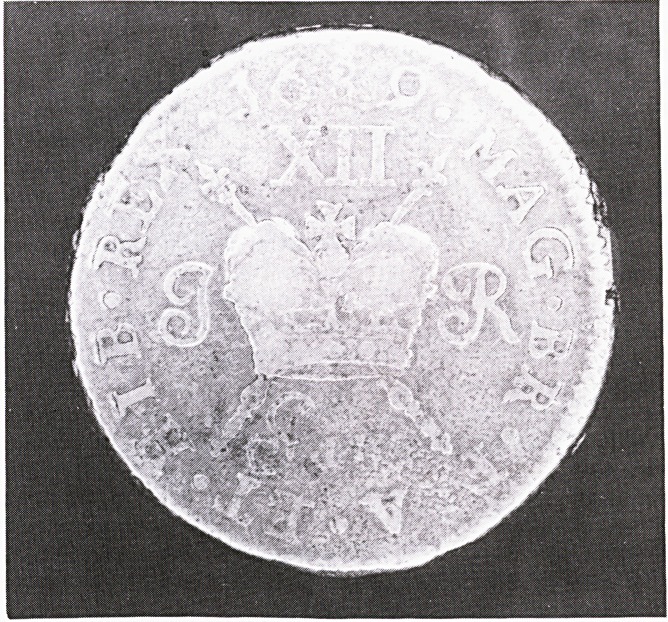# Gun Money, Owning a Piece of History

**Published:** 1986-04

**Authors:** Paul Goddard


### Gun Money—Owning a piece of history

**By Paul Goddard**

James the second had a short and unhappy reign. Whilst his brother Charles had been intelligent and charming James had none of Charles brain and still less of his charm.

In the summer of 1685, James II overcame invasions in the north and in the south. The invasion in the south had been led by the Duke of Monmouth, illegitimate son of Charles the second. Monmouth had landed in Dorset and had been immediately proclaimed King by his supporters. He had, however, obtained no following in Bristol or in Wiltshire. Monmouth was defeated in a battle at Sedgemoor and, riding for his life, he was taken in Cranborne Chase. After a pitiful interview with the King he was executed. The following reprisals were sickening because of their viciousness. Chief Justice Jeffreys carried out his 'bloody assize' which made the West country a shambles and in which some three hundred prisoners were executed and hundreds more transported to the Indies.

James II went on to demand a larger standing army and he sought greater power for Catholics. In 1687 he dissolved parliment.

Meanwhile William of Orange, the husband of James's daughter Mary and a staunch Dutch protestant, was preparing to come to England. The glorious revolution started with a landing at Torbay in early November 1688 and within two months William had routed the King's forces and James was obliged to flee to France.

James was not however happy to give up the crown without a fight and he returned to Ireland with considerable support and that country was soon plunged into turmoil. In mid 1689 James was running out of money to pay his army. His first step was to increase the value of English coin in circulation and to double the value of the French 3½ sou pieces. But more coins were needed urgently and he thus established two mints, one at Dublin and the other at Limerick. He instructed his agents to obtain brass and all other metals suitable for coinage and by Royal proclamation reminded the general public that punishment awaited anyone who refused to accept the base metal coins. The coins were made from material such as old church bells, kitchen utensils and disused cannons. The melting down of the cannons led to the name Gun Money.

While the metal content left much to be desired the workmanship was remarkably good due to the excellent engraving of Jan Roettier. A Gun Money shilling is shown here (Figures 1 and 2). Initially the shillings and half crowns were struck on flans of approximately the same size as corresponding English denominations but when metal became increasingly difficult to obtain smaller pieces were struck.

William III defeated James at the Battle of the Boyne in July 1690. Once more James made his way to France where he died in 1701.

After the defeat of James, William immediately devalued the gun money coins stating that the large shilling was only worth a half penny and the smaller variety a farthing. Within a short time the Gun Money was prohibited altogether resulting in considerable hardship and misery for the population. Gun Money coins still remain popular with collectors almost two hundreds years after their withdrawal from circulation in Ireland. The coin shown here would be worth about £10.00 and considering its fascination as a piece of history and as a conversation piece such coins make a good investment despite their base metal content.

### REFERENCES

1. The Splended Shilling by James O'Donald Mays.
2. A History of England by Keith Feiling.

## Figures and Tables

**Figure f1:**
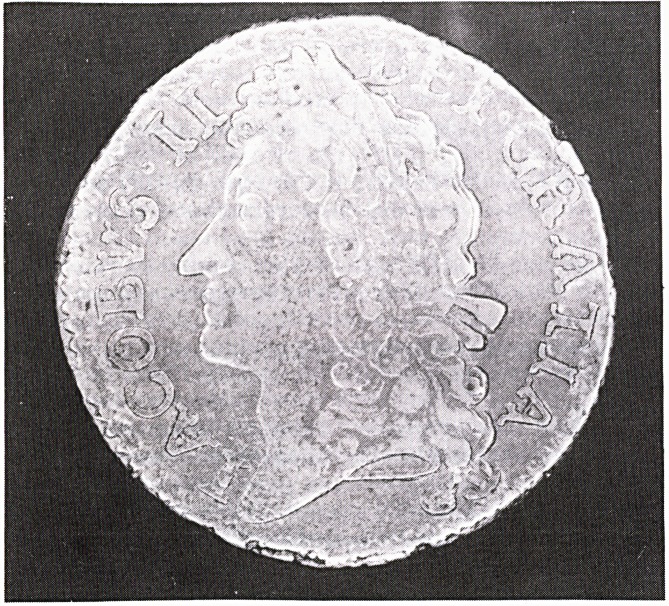


**Figure f2:**